# Examination of Athlete Triad Symptoms Among Endurance-Trained Male Athletes: A Field Study

**DOI:** 10.3389/fnut.2021.737777

**Published:** 2021-11-26

**Authors:** Erin M. Moore, Clemens Drenowatz, David F. Stodden, Kelly Pritchett, Thaddus C. Brodrick, Brittany T. Williams, Justin M. Goins, Toni M. Torres-McGehee

**Affiliations:** ^1^Kinesiology Department, University of Virginia, Charlottesville, VA, United States; ^2^Division of Sport, Physical Activity and Health, Linz, University of Upper Austria, Upper Austria, Austria; ^3^Physical Education, University of South Carolina, Columbia, SC, United States; ^4^Health Sciences, Central Washington University, Ellensburg, WA, United States; ^5^Exercise Science, Exercise Science Department, University of South Carolina, Columbia, SC, United States

**Keywords:** low energy availability, bone mineral density, testosterone, reproductive dysfunction, macronutrients, male endurance athletes, male athlete triad in endurance athletes

## Abstract

**Background:** Studies examining the physiological consequences associated with deficits in energy availability (EA) for male athletes are sparse.

**Purpose:** To examine male athlete triad components; low energy availability (LEA) with or without an eating disorder risk (ED), reproductive hormone [testosterone (T)], and bone mineral density (BMD) in endurance-trained male athletes during different training periods.

**Methods:** A cross-sectional design with 14 participants (age: 26.4 ± 4.2 years; weight: 70.6 ± 6.4 kg; height: 179.5 ± 4.3 cm; BMI: 21.9 ± 1.8 kg/m2) were recruited from the local community. Two separate training weeks [low (LV) and high (HV) training volumes] were used to collect the following: 7-day dietary and exercise logs, and blood concentration of T. Anthropometric measurements was taken prior to data collection. A one-time BMD measure (after the training weeks) and VO_2max_-HR regressions were utilized to calculate EEE.

**Results:** Overall, EA presented as 27.6 ± 10.7 kcal/kgFFM·d-1 with 35% (*n* = 5) of participants demonstrating increased risk for ED. Examining male triad components, 64.3% presented with LEA (≤ 30 kcal/kgFFM·d-1) while participants presented with T (1780.6 ± 1672.6 ng/dl) and BMD (1.31 ±.09 g/cm^2^) within normal reference ranges. No differences were found across the 2 training weeks for EI, with slight differences for EA and EEE. Twenty-five participants (89.3%) under-ingested CHO across both weeks, with no differences between weeks.

**Conclusion:** Majority of endurance-trained male athletes presented with one compromised component of the triad (LEA with or without ED risk); however, long-term negative effects on T and BMD were not demonstrated. Over 60% of the participants presented with an EA ≤ 30 kcal/kgFFM·d-1, along with almost 90% not meeting CHO needs. These results suggest male endurance-trained athletes may be at risk to negative health outcomes similar to mechanistic behaviors related to EA with or without ED in female athletes.

## Introduction

Examination of low energy availability, defined as <30 kcal/kg^−1^FFM·d^−1^ and the associated physiological ramifications is no longer only examined in females in sports research. Recent literature has demonstrated similar physiological consequences [decreased reproductive hormones and bone mineral density (BMD)] in males, predominately in endurance athletes (i.e., runners, cyclists and triathletes) (1–5) due to LEA (1–3, 5, 6). Due to high exercise energy expenditure (EEE) demands of endurance sports, male endurance athletes are at an increased risk of LEA, decreased testosterone (T), and low BMD (6) due to the nature of their sports, which includes massive EEE and inadequate energy intake (EI) compensation. Recent research has demonstrated negative physiological responses with LEA and hormonal changes in males (7). Two potential manifestations of decreased T in males include an acute response due to LEA and/or excessive training loads or a chronic response labeled exercise-hypogonadal male condition (EHMC) (8). Understanding the hormonal responses to LEA in male athletes is crucial for overall health and successful performances (i.e., illnesses/injuries).

A recently established working model for male athletes has demonstrated similar syndrome characteristics as demonstrated in the female athlete triad (Triad) (2). The triad is composed of three components: LEA with or without an eating disorder (ED), hypothalamic reproductive dysfunction, and compromised BMD (9). Symptoms for males include metabolic and endocrine changes (decreased leptin, insulin-like growth factor-1 GF-1, and increased cortisol) due to decreased EA, decreased T, libido and sperm quality, decreased BMD and bone markers, and increased risks of bone stress injuries (2). Clinically relevant biomarkers [reproductive hormones (luteinizing hormone, estrogen, and follicular stimulating hormone), bone mineral density, and bone hormone markers] have been used to assess LEA and the health of female athletes regarding physiological consequences that occur at and below an EA of 30 kcal/kg^−1^FFM·d^−1^ and exist for females (9). These biomarkers have not been established for the physically active male, leading the female athlete triad coalition and the International Olympic Committee (IOC) to identify males as an under-researched population (4, 9). Due to the recognized physiological differences between males and females, necessity to establish independent clinical guidelines for males in regard to LEA and their collective metabolic and physiological impacts has been stressed (10). Parallels between triad symptoms in males and females and have been determined, and a better understanding of nutritional deficits is needed for males, including a definition of LEA for males, confirmation whether disordered eating (DE)/ ED risk contributes to chronic LEA, and the relationship of EA to metabolic and physiological changes (1).

Our study sought to address this need by examining male triad symptoms (EA with or without ED risk, T, and BMD) among endurance trained male athletes. We hypothesized that endurance trained athletes would display at least one compromised component of the male athlete triad. A secondary purpose was to examine differences in energy needs (e.g., EI, EEE, and EA,) and hormonal changes (T) across 2 separate training volume weeks [high volume (HV) training week and low volume (LV) recovery week]. We hypothesized male endurance-trained athletes would display significant difference in energy needs between the 2 training weeks.

## Materials and Methods

### Experimental Design and Participants

This study implemented a within-subject cross-sectional design examining recreational male endurance athletes (distance runners, triathletes, and obstacle racers), their energy needs (EA, EEE, and EI), T, and BMD. Fourteen male participants (age: 26.4 ± 4.2 years; weight: 70.6 ± 6.4 kg; height: 179.5 ± 4.3 cm) were recruited from a local community in the Southeastern region of the United States. Inclusion criteria were: (a) actively training and competing ≥10 h/week for at least 3 months (11, 12) within a competitive season, (b) body fat percentage (BFP) ≤ 12%, (11, 12) (c) maintained weight stability (±3 kg in past 6 months) (13), and d) had a VO_2max_ considered excellent for age-specific range. The VO_2max_ treadmill test using the method from Beashel et al. (14) targeted for endurance runners was administered prior to data collection. This test utilized a continuous speed (7 mph) with graded % incline (14). Exclusion criteria included past or present diagnosis of clinical ED, history of cardiovascular disease, thyroid, pituitary or other metabolic disease or orthopedic impairment that interferes with moderate to vigorous exercise. Institutional Review Board approval was obtained, and all the participants provided written consent.

### Instruments/Protocols

#### Anthropometric Measurements and DXA

Basic demographic information and anthropometric measurements were collected according to ACSM standardized procedures (15). Body fat was assessed using Tanita scale (Tanita SC-331S Body Composition Scale, Tanita Co., Tokyo, Japan) for an inclusion criterion and Dual-Energy X-Ray Absorptiometry (DXA) (GE Lunar Prodigy densitometer) for data analysis and to measure BMD (g/cm^2^) of the total body (16).

#### Energy Assessment

Multiple measures were assessed (EI and EEE), measured [resting metabolic rate (RMR)], and calculated [EA and energy balance (EB)] in this study. Indirect calorimetry *via* MedGem Analyzer (*MicrolifeMedGem*; HealthTech, Golden, CO) protocol, utilizing a mouthpiece and nose plug, was used to measure RMR at the beginning of data collection. Even though the MedGem is not the gold standard, it is a clinically validated RMR measurement device (17). To assess EI, the participants recorded their food and fluid intake (estimated) for 7 consecutive days during 2 separate weeks. Visual aids assisted subjects in entering portion sizes prior to recording into the ESHA food processor (ESHA food processor 8.0, Salem, OR). Dietary records were analyzed for total kilocalories (kcals), macronutrient and micronutrient consumption using a dietary analysis software program. Goldberg ratio was calculated for both the HV week (1.52) and LV weeks (1.49) to examine validity of EI reports (18). Individual VO_2max_ –HR regression slopes were used to calculate EEE *via* exercise logs and HR monitors (11). Energy outcomes were measured *via* energy balance (EB) and EA. Energy balance, defined as the total daily energy expenditure (TDEE), was measured with Bodymedia Sense Wear Armbands, and EI remaining at an equal level {EB = [EI (kcals/day) = TDEE kcal/day)]}. Energy availability, defined as the amount of dietary energy remaining after exercise, expressed as kcal/kg/free fat mass [EA = (EI–EEE) kcal/kgFFM·d^−1^], was both examined (9).

#### Eating Disorder Risk

Risk of an ED was evaluated using the Eating Disorder Inventory-3 (EDI-3) and Symptom Checklist (EDI-3 SC). The EDI-3 is a screening tool designed specifically for Allied Health professionals to identify individuals at risk for ED while the EDI-3 SC provides information about the frequency of ED pathogenic behaviors (19). The EDI-3 is a self-reported survey (validated for males and females aged 13–53) that identifies subjects with DE patterns (19). Reliability is high with coefficient and median values for specific composites: ED risk (*r* = 0.98, median =0.95) and General Psychological Maladjustment (*r* = 0.97, median = 0.93) (19). To be determined “at risk” for ED, the participants must be identified as “typical clinical” or “elevated clinical” for at least 1 EDI composite score, and/or meet the criteria for risk of pathogenic behavior (19).

#### Blood Sampling and Storage

Blood samples were collected for both weeks between 0,530 and 0,730 h during the 8th day of the weekly protocol. The participants were instructed to refrain from exercise for 24 h and consumption of food 12 h prior to blood draws. All OSHA guidelines were followed to minimizing any exposure risks to the participants. Using 21G (19 mm) BD vacutainer needles and tubes (BD Vacutainer; Becton, Dickinson and Company, Franklin Lakes, NJ), samples were taken from the antecubital vein, centrifuged (Eppendor Centrifuge 5702F) for 15 min at 4°C and pipetted into 2-ml polyethylene tubes for storage in a −80°C freezer for 1 month prior to analysis. Blood samples were assessed using enzyme-linked immunosorbent assay (ELISA) kits to measure testosterone levels. Sensitivities of ELISAs are high, 1–10 ug/liter range with correlation coefficients ranging between 0.95 and 0.99 (20), specific T ranges 1–18 ng/ml.

#### Training Categorization

**Figure 1:**
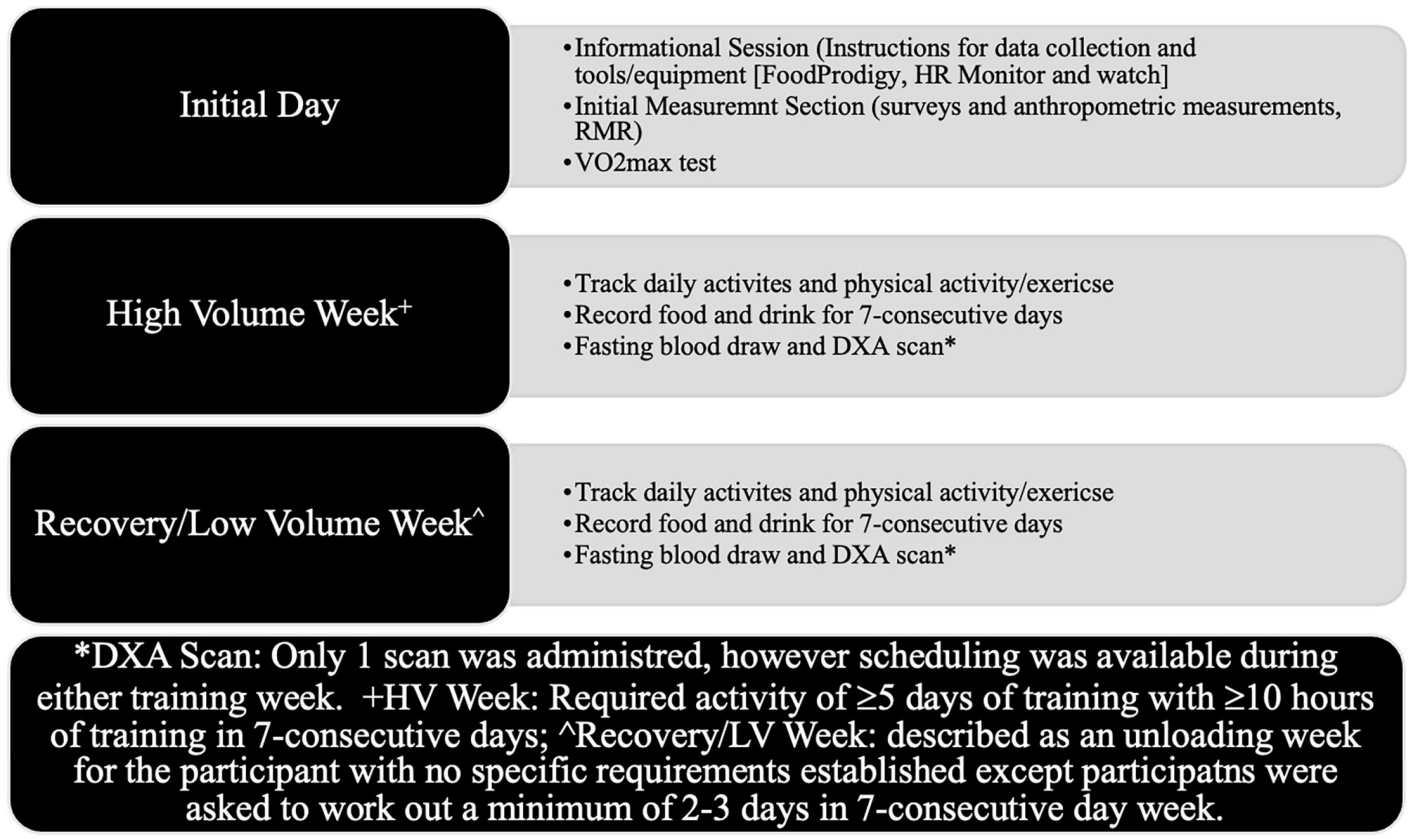
Data collection protocol.

Differences in energy needs and T were assessed during two training weeks with different intensities. High-volume training week (HV) consisted of >5 days of training with >10 h of training in a 7-consecutive-day week and a low-volume training week (LV) described as an unloading week for the participant with no specific requirements established except the participants were asked to work out a minimum of 2–3 days. Specific protocols for data collection can be found in Figure 1.

### Statistical Analysis

Specific cut point measures were defined for multiple variables, including EA where LEA defined as <30 kcal/kg FFM·d^−1^, while EB was defined as EB [EI = TDEE], negative EB [EI < TDEE] or positive EB [EI > TDEE] (9). Testosterone cutoffs were based on previously established normative data specific for males, T = 270–1070 ng/dl (21). Testosterone was identified as low, within normal limits, or high based on normative data. A Z-score >-0.9 was considered within the normal range (16, 22).

IBM SPSS statistical software (version 26; SPSS Inc., Armonk, NY) and an *alpha* ≤ 0.05 were used for all analyses. Based upon power analysis *a priori* and based upon means of previous literature from Koehler et al. (13) and Loucks et al. (23), an effect size between 1 and 3 yielded a sample size of 6–10 subjects. Using the Wilcoxon-signed rank test, 14 subjects were allowed for full saturation. Descriptive statistics were calculated for all dependent variables. Frequencies and proportions with 95% confidence intervals (CI) were calculated for all categorical variables. Crosstabulations and chi-square analysis were used to examine “at risk” variables, while macro/micronutrient profiles were compared to ACSM recommendations. A 2-(week)-X7 (days) ANOVA and paired *t*-tests assessed differences between the training weeks for TDEE, EB, EA, EI, EEE, and macro/micronutrients. Pearson's correlations and regressions were used to examine relationships between T, LEA, and other continuous variables.

## Results

Eighteen participants began the study, three were eliminated for lack of compliance with study procedures, and one dropped out due to fear of needles, yielding a total of 14 participants. Demographic information, energy needs assessment, inclusion criterion, and macro/micronutrient intakes are reported in Tables 1–5.

**Table 1:** Basic demographics and an inclusion criterion for endurance trained male athletes (*n* = 14).

	**ALL**	
**Basic demographics**	**M**	**SD**
Age (years)	26.4	4.2
Height (cm)	179.5	4.3
Weight (kg)	70.6	6.4
BMI (kg/m^2^)	21.9	1.8
**Ethnicity**	**N**	**%**
African American	2	14.3
Caucasian	11	78.6
Middle Eastern/Kurd	1	7.6
**Education level**		
High School Diploma/GED	1	7.1
Attained some level of college	4	28.6
Bachelor's Degree	3	35.7
Master's Degree	5	35.7
Clinical Doctorate	1	7.1
**Inclusion criterion**	**M**	**SD**
VO_2max_ (ml/kg/min)	62.3	6.9
Free Fat Mass (kg)	65.7	5.4
Tanita BFP (%)	7.1	2.2
DXA BFP (%)	13.6	3.5

*Values are presented in mean ± standard deviation*.

**Table 2:** Energy needs assessment and male athlete triad components for endurance-trained male athletes (*n* = 14).

**Energy needs assessment**	**M**	**SD**	***p*** **-value**	
Resting Metabolic Rate (kcals)	1,799	549		
Exercise Energy Expenditure (kcals)	865	566	*p* = 0.13	
HV EEE (kcals)	1,048	805		
LV EEE (kcals)	682	326		
Energy Intake (kcals)	2,658	887		
HV EI (kcals)	2,687	878		
LV EI (kcals)	2,629	927	*p* = 0.18	
			*p* = 0.31	
Energy Availability (kcal/kgFFM·d)	27.6	12.1	*p* = 0.13	
HV EA (kcal/kgFFM·d)	25.2	12.9		
LV EA (kcal/kgFFM·d)	29.9	11.1		
Testosterone (ng/dL)	1,780	1,672	*p =* 0.28	
HV T (ng/dL)	1339	836		
LV T (ng/dL)	1455	889		
Bone Mineral Density (g/mc^2^)	1.3	0.9	*p* = 0.34	
Overall Distance (miles)	49.1	77.9	*p* = 0.08	
HV Distance (miles)	63.4	100.6		
LV Distance (miles)	34.9	45.4		
Overall Training Time (hours)	5.6	4.3	*p* = 0.06	
HV Training Time (hours)	7.1	5.3		
LV Training Time (hours)	4.1	2.5		
**Low energy availability risk**	**HV%**	**N**	**LV%**	**N**
LEA <2 days per week–VO_2_	35.7	5	50	7
LEA 3–4 days per week–VO_2_	35.7	5	21.4	3

*Values are presented in mean ± standard deviation. LEA, low energy availability; EEE, exercise energy expenditure, calculated by the average number of days of exercise/week across individuals*.

**Table 3:** Eating disorder characteristics among endurance trained male athletes (*n* = 14).

	**EDI classification**							
	**Raw score**		**Low clinical**		**Typical clinical**		**Very typical**	
	**Mean**	**SD**	***n***	**%**	***n***	**%**	***n***	**%**
**Eating Disorders Risk Scale**								
Drive for thinness (DT)	1.6	2.4	14	100	–	–	–	–
Bulimia (B)	0.9	1.9	13	92.9	**1**	**7.1**	–	–
Body dissatisfaction (BD)	1.6	2.4	14	100	–	–	–	–
Eating disorder risk composite (EDRC)	82.3	5.8	14	100	–	–	–	–
**Psychological scale**								
Low self-esteem (LSE)	2.2	3.8	13	92.9	**1**	**7.1**	–	–
Personal alienation (PA)	1.9	2.3	14	100	–	–	–	–
Interpersonal Insecurity (II)	4.9	4.4	11	78.6	**3**	**21.4**	–	–
Interpersonal alienation (IA)	3.4	3.2	10	71.4	**4**	**28.6**	–	–
Interceptive deficits (ID)	0.8	1.1	14	100	–	–	–	–
Emotional dysregulation (ED)	0.6	0.8	14	100	–	–	–	–
Perfectionism (P)	10.1	4.3	6	42.9	**7**	**50**	**1**	**7.1**
Asceticism (A)	5.4	3.8	11	78.6	**3**	**21.4**	–	–
Maturity fears (MF)	7.1	5.8	6	42.9	**5**	**35.7**	**3**	**21.4**
**Composite**								
Ineffectiveness composite (IC)	63.1	9.6	13	92.9	**1**	**7.1**	–	–
Interpersonal problems composite (IPC)	76.3	11.5	11	78.6	**3**	**21.4**	–	–
Affective problems composite (APC)	66.6	2.1	14	100	–	–	–	–
Over control composite (OC)	80.6	12.4	10	71.4	**4**	**28.6**	–	–
General psychological maladjustment (GPMC)	331.1	30.5	14	100	–	–	–	–

*Data are presented in frequency (n) and percent (%). Bold values signify findings of significant values of note*.

**Table 4:** Eating disorder pathogenic behaviors among recreational endurance-trained male athletes (*n* = 14).

	**All data**	
**Exercise to control weight**	**N**	**%**
0% of time	8	57.1
<25% of time	**4**	**28.6**
25–50% of time	**2**	**14.3**
More than 75% of time	0	0
100% of time	0	0

*Data are presented in frequency (n) and percent (%). Bold values signify findings of significant values of note*.

**Table 5:** Daily energy, macro and micronutrient intake in the high- and low-volume training weeks (*n* = 14).

	**Overall (*****n*** **= 14)**		**High volume**		**Low volume**		
	**M**	**SD**	**M**	**SD**	**M**	**SD**	***p*** **-value**
Energy Intake (kcals)	2,658	887	2,687	878	2,629	927	0.18
CHO (g/kg)	4.9	1.7	4.6	0.5	4.7	0.5	0.7
PRO (g/kg)	1.7	0.6	1.7	0.2	1.7	0.1	0.28
Fats (% of kcals)	32.3	5	31.6	1.7	31.5	1.4	0.95
Vitamin B_6_ (mg)	2.4	1.2	1.2	0.9	2.7	1.2	0.09
Vitamin B_12_ (mcg)	6.3	3.6	6.4	3.3	6.3	3.9	0.91
Calcium (mg)	1206.2	653	1222.5	621.9	1189.9	705.9	0.9
Iron (mg)	23.9	10.5	24.7	11.1	23.1	10.3	0.69

*Means and standard deviation are reported for both training weeks. p-value equal to or < 0.05 will indicate a significant difference*.

### Male Athlete Triad

Overall, no participants met the criteria for all three male triad components, and the participants did not present at risk for either low T levels or compromised bone health. Results demonstrated 64.3% (*n* = 9) of the participants presented with LEA over the 2 training weeks, and, of those, 60% (*n* = 3) presented as LEA with an ED risk. Overall, 35.7% (*n* = 5) of the participants presented at risk for EDI-3 composite scales (see Table 3). One participant did demonstrate a typical clinical score for Bulimia (7.1%). One specific composite, maturity fears, presented with the highest scores between all the sub-scales with 35.7% (*n* = 5), demonstrating typical clinical scores and 21.4% (*n* = 3) with elevated clinical scores.

### Energy Needs

No differences were found across the 2 training weeks for EI (see Table 4). Slight differences were elicited for EA across the training weeks (HV: 25.2 ± 12.9 kcal/kg FFM·d^−1^ vs. LV: 29.9 ± 11.1 kcal/kg FFM·d^−1^, *p* = 0.13. This decrease was not due to a change in EI but rather to a difference noted in EEE with the LV week eliciting lower EEE kcals (HV: 1,048.5 ± 805.6 kcals/week vs. LV: 682.3 ± 326.5 kcals/week, *p* = 0.13) (see Table 4). Carbohydrate intake was low with 89.3% (*n* = 25; HV: 92.9%, *n* = 13, LV: 85.7%, *n* = 12) of participants consuming <5 g/kg of CHO across both training weeks. Due to missing data cells (determined by the days the participants exercised), a paired samples *t*-test was calculated to compare the mean of the training weeks. No significant differences were elicited between the weeks (t_(13)_ = 1.7; *p* = 0.10). Average TDEE of the participants for both weeks was 2,993 ± 160 kcal, which resulted in an overall negative EB of −39 ± 201 kcal. There was no significant main effect found between the training weeks and TDEE: F_(1, 11)_ = 4.02 (*p* = 0.07) or EB: F_(1,13)_ = 4.40, (*p* = 0.06).

### Testosterone

Mean T levels between the 2 training weeks can be found in Table 2. A negative correlation was found between overall T levels to EI: (r_(26)_ = −0.47, *p* = 0.02), RMR: (r_(26)_ = −0.64, *p* < 0.001), while a positive correlation for overall T levels to DXA_BFP [r_(26)_ =0.83, *p* < 0.001]. Two outliers, determined as more than two standard deviations, were removed prior to regression analysis. The regression equation for T and EA was not significant [F_(1,23)_ = 3.2, *p* = 0.89].

## Discussion

This study aimed to examine all three components of the male athlete triad observed in free-living subjects. Our overall results supported our hypothesis that recreational endurance-trained male athletes would exhibit >1 component of the male triad. We found 64.3% (*n* = 9) exhibited LEA (with or without an ED risk); however, our participants did not demonstrate decreased T or BMD. Examination of LEA with or without ED may be prudent to understand mechanistic behaviors that affect T and BMD in males and assisting in defining a set point for LEA.

### Energy Availability

Within this study, 2 separate training weeks (HV and LV) were used to examine energy needs (i.e., EA with or without ED, EI, EEE, TDEE, and EB). Our results yielded an average EA of 27.6 kcal/kg FFM·d^−1^, similar to the results reported by Hooper et al. (7), which examined nine long-distance runners who presented with exercise-hypogonadal male condition eliciting EA levels of (27.2 ± 12.7 kcal/kg FFM·d^−1^). Lane et al. (24) also found similar levels in cyclists (26.9 ± 17.4 kcal/kg FFM·d^−1^) and in non-elite endurance athletes (28.7 ± 13.4 kcal/kg FFM·d^−1^) (25).

The results from the present study indicated high prevalence of LEA compared with three studies resulting with prevalence rates at 23 (26), 42 (27), and 54% (28) of male cross-country runners. Within our participants, we found an increase in EEE during the HV training week, while EI remained stable between the weeks. This was demonstrated by Woods et al. (29), which exhibited no significant changes in EI related to different training blocks in cyclists. Viner et al. (30) found small difference in cyclists between preseason (18.8 ± 12.2 kcal/kg FFM·d^−1^), competitive season (19.5 ± 8.5 kcal/kg FFM·d^−1^), and off season (21.7 ± 9.2 kcal/kg FFM·d^−1^), EA levels and EI during preseason (29.3 ± 6.8 kcal/kgbm·d^−1^) competition season (34.7 ± 6. kcal/kgbm·d^−1^) and off-season (31.8 ± 7.5 kcal/kgbm·d^−1^). This is concerning as LEA is the catalyst for negative physiological functions associated with the triad (9). Koehler et al. (13) and Loucks et al. (23) have demonstrated LEA is elicited with or without exercise, which emphasizes the importance of proper nutritional practices throughout training protocols. Currently, there is no clear LEA cutoff for males. In this study, we found an EA level ranging between ~25 and 29 kcal/kg FFM·d^−1^ during the 2 training weeks, which did not demonstrate negative T levels or BMD. This provides support to the belief that males may sustain lower EA levels and maintain functioning metabolic and endocrine systems. More research targeted between 15 and 25 may be warranted, as Koehler et al. (13) found metabolic changes (Leptin and IGF-I) at 15 kcal/kg FFM·d^−1^.

In the present study, EI was not statistically different between the training weeks, suggesting this group of recreational male endurance-trained athletes are not changing their EI in relation to the demands of training volumes. While EI derived from the food frequency questionnaires demonstrated similar intakes (2,623 ± 796 kcals) in male ultramarathon runners (7), EI was lower when compared to mountain runners (3,199 ± 701 kcal/day) prerace-day diet (31) and non-elite endurance athletes (3,073 ± 777 kcals/day) (25). The absence of fueling changes between training weeks could be contributed to a singular or combination of mechanisms, including a lack of nutritional knowledge related to fueling needs of different training volumes, possible pathogenic eating, and feeding behaviors, or, perhaps, metabolic signals are being interrupted. One possible area contributing to LEA includes carbohydrate (CHO) intake. Levels were lower than the recommended intake for endurance athletes ranging from 5–10 g/kg, depending on activity levels. This is congruent with other studies that examined cyclists, runners, and triathletes ranging from 3.9 ± 1.2 g/kg (30) to 4.8 ± 1.5 g/kg/day (26). This suggests that recreational endurance athletes are under fueling or restricting CHO aids in LEA and potentially demonstrating pathogenic eating behaviors.

The participants in this study demonstrated either similar or lower values of EEE (~870 kcals) compared to data from other studies with males, including army rangers (~4,000 kcal/day) (12), race days for professional jockeys (~3,952 kcal) (32), cyclists, runners, and triathletes [1,047 ± 718 kcals/day (30) [914.1 ± 143.5 kcals] (7), [760 ± 404 kcals/day] (26), 1,296 ± 466.7 kcals/day] (25). This decrease in EEE kcals may be due to the sample characteristics of the different studies. Military operation studies required significant EEE demands placed on soldiers compared to self-selecting workouts of the participants of this study. Variations in calculations and definitions of EEE could be a contributing factor to differences found within EEE to other male studies (33) as well as athletic elite status (i.e., professional vs. recreational athletes).

### With or Without an Eating Disorder

Overall, 35.7% presented with an increased ED risk with 7.1% specific to Bulimia subscale. This percentage is elevated compared with previous, albeit limited research on male athletes (34–36). Research has estimated 10% of elite male athletes present with anorexia nervosa and 25% present with bulimia nervosa, (36) while another 1% of males represent “other specified feeding or eating disorders” (37). Norwegian researchers found athletes (13.5%) were two times as high to be diagnosed with a clinical and/or subclinical ED as non-athletes (4.6%) (35). Sundgot-Borgen et al. (35) also demonstrated 9% of endurance athletes presented with diagnosed ED. Athletes with increased rates of pathogenic behaviors, along with the underrepresented prevalence and rates in male athletes, are of paramount concern due to effects of pathogenic behaviors on fueling and EA (6, 35).

The EDI-3 has specific composite and sub-scale scores, which examine specific behavioral traits similar to those diagnosed with EDs, with “typical clinical” and “elevated clinical” scores, indicating increased risk factors in EDs. Two specific psychological risks identified in the over-control composite, which demonstrated 57.1%, were classified as typical/elevated clinical for perfectionism and maturity fears. The raw scores for perfectionism (10.1 ± 4.3) were similar to scores of female athletes, including equestrian: 13.2 ± 5.8, volleyball: 10.8 ± 4.3, softball: 15.1 ± 5.3, beach volleyball: 9.9 ± 4.8, and soccer: 12.7 ± 4.7 (38). The over-control composite reflects the significant need to avoid disappointing others as well as being the best. The sub-scale perfectionism is important to note, as this sub-scale is a distinguishing feature of EDs as well as strivings in other areas such as athletics (19). This sub-scale demonstrated in this study was over 50% of our participants demonstrating typical/elevated scores.

Pathogenic eating behaviors presented included using exercise for weight control. Research suggests 37% of male athletes (age, 18–22) exercise 2 or more hours per day for the purposeful intention to burn calories (39). With 42.9% of the participants using exercise to lose weight up to 50% of the time, this presents concern as to the motives of males exercising, whether for health benefits (i.e., cardiovascular fitness) or due to risks of ED behaviors. Torres-McGehee et al. (38) found 38% of female athletes, and performance artists also engaged in additional exercise to control their weight. Similar to the female literature, elite male athletes participating in sports that require leanness (endurance athletes) show increases in ED risk (37). However, to date, the literature focused on EDs is limited, and, currently, the ability to compare male athletes to other male control groups is currently not available (37). More research is needed for male athletes regarding pathogenic eating and feeding behaviors.

### Energy Balance

Endurance-trained male athletes demonstrated negative EB (−289.4 kcal) in the HV training week, which elicits concern as this is indicative of poor fueling to meet the demands of TDEE. Prolonged negative EB and LEA lead to decreased physiological processes (i.e., lower RMR); (9) however, the disparity among EB and EA is that an athlete can have LEA but maintain their EB. This is due to the suppression of various physiological processes due to the lack of EA (9). Strubbs et al. (40) provided an example of the contrast between EB and EA, using eight lean men with suppressed caloric EI and an increased EEE, resulting in a constant EA of 30 kcal/kg FFM·d^−1^. Additionally, they found that negative EB decreased toward zero at a rate of 90 kcal·d^−1^ due to the decreased physiological processes and estimated 3 weeks for the participants to elicit an EB of zero while stilling remaining in a LEA state (40). Thus, confirming EI intake was inappropriate for not just exercise but also the daily living of subjects.

### Testosterone

Both weeks demonstrated values within or greater than the “normal” range of 270–1,070 ng/dL. Regression results demonstrated a poor predictor of T levels. With the average for both weeks demonstrating high levels of T, this was not congruent with the literature examining endurance runners (3, 5, 7, 41). Testosterone is considered a more robust hormone, with delays in response to external stimuli (i.e., decreased body fat and increased mileage) (42). One theory is the “volume threshold” where decreases in T are elicited when participants are trained at >100-km^.^week^−1^ (43). This threshold was congruent with our participants during the HV week for distance but did not demonstrate decreases in T. Our results are congruent with Koehler et al. (13), who examined six cyclists and did not find a decrease in T when EA was acutely reduced to 15 kcal/kg FFM·d^−1^ for 4 days. Of note, all the participants participated in weightlifting programs along with their endurance training. This was not a requirement for inclusion but was tracked for EEE/TDEE of training. While literature has demonstrated acute increases in androgen responses in males during weight lifting, chronic adaptations have not been expressed (44). Long-term weight lifting of the participants may be attributed to the lack of decreases in T. Also, variables may not have been stressed enough to elicit decreases in T, including EA (~27 kcal/kg FFM·d^−1^) may not be low enough, body fat percentages (~13.5%) may need to be less, EEE (~670 kcals) may need to be increased, and consistent increased mileage (~101 km) increased.

### Bone Mineral Density

Zero participants demonstrated low BMD; however, 29% (*n* = 4) participants ranged from −0.4 to 0.9, including an African-American participant with a BMD at −0.4, which was surprising due to African-Americans present with more dense bone in comparison to Caucasians (45). Two participants were well-over a Z-score of 2; these were obstacle course runners and, therefore, did large amounts of weight lifting along with endurance running. Many participants were active in a weight lifting regimen, which may be related to the adequate BMD levels. Finally, due to mechanical limitations, segmental DXA scans were not available, and, in the future, specific sites, including spine and femur, should be examined.

### Limitations and Future Research

There were limitations identified in this study. First, while we did meet power, our sample size was still low (*n* = 14). Second, while we used a crossover design, using a separate control group for future studies should be considered. Third, EI collection was a self-reported measurement and, with all self-reported measurements, could have errors, underreporting of non-nutritious foods and over reporting of nutritious foods (46); however, research has demonstrated that, despite food intake restrictions, reported intake accuracy was superior using a 7-consecutive-day diet record compared to a 3-day recall (47). More accuracy may be obtained using a ventilation hood for RMR data. Examination of EEE was calculated using VO_2max_-HR regression due to the field nature of the study; double-labeled water is still the gold standard for accurate EEE, whereas measuring EEE by indirect calorimetry during exercise may decrease underestimation of EEE for field studies (48). Regarding potential errors in calculations of EI and EEE, Burke et al. (49) recommend development of a standardized measurement format for future work in triad and relative energy deficiency. In achieving due to the large normative range of the hormone T, establishing a “normative range” specific to male athletes is needed regarding identifying long-term testosterone responses to various training variables (i.e., running and strength training). Future research should examine reproductive hormones more specifically in relation to LH and FSH to examine the pulsatile nature of the response of the hormone, and possibly examining a sperm count to assess the output measures of LH and T in males. Future research should begin to look at and address subclinical T levels (~50–75%) and establish a consistent unit of measure for T across studies for consistency.

## Conclusion

Our study demonstrated 64.3% recreational endurance-trained males presented with one compromised component of the triad (LEA with or without ED), with participants demonstrating the use of pathogenic behaviors. Further research is needed to examine specific nutritional behaviors of male endurance athletes, including the restriction of CHO intake and pathogenic behaviors related to EDs, EA with or without EDs, and the corresponding physiological health markers, including metabolic hormones (Leptin, Insulin, Cortisol) and bone markers (procollagen type 1 N-terminal propeptide, C-terminal telopeptide type 1 collagen) in male athletes. A more accurate range of T levels also needs to be established in relation to male athletes *vs*. the general population. While females present physiological issues at an EA level of 30 kcal/kg FFM·d^−1^, this study and others have demonstrated set points of males may be lower. Thus, more research on the mechanistic nature of the triad (decreased EA with or without ED, reproductive hormones, and BMD) needs to be assessed and established in the male athlete population. Currently, there is no set cutoff point for LEA in the male population or a clear understanding of physiological consequences for males regarding triad symptoms. More research is needed for the overall metabolic and nutritional health and performance for these athletes.

## Data Availability Statement

The raw data supporting the conclusions of this article will be made available by the authors, without undue reservation.

## Ethics Statement

The studies involving human participants were reviewed and approved by University of South Carolina Institutional Review Board. The patients/participants provided their written informed consent to participate in this study.

## Author Contributions

This study was designed by EM, TT-M, CD, DS, and JG. Data were collected and analyzed by EM, TT-M, TB, BW, and KP. Data interpretation and manuscript preparation were undertaken by EM, TT-M, TB, BW, CD, KP, DS, and JG. All authors approved the final version of the paper. The results of this study are presented clearly, honestly, and without fabrication, falsification, or inappropriate data manipulation.

## Conflict of Interest

The authors declare that the research was conducted in the absence of any commercial or financial relationships that could be construed as a potential conflict of interest.

## Publisher's Note

All claims expressed in this article are solely those of the authors and do not necessarily represent those of their affiliated organizations, or those of the publisher, the editors and the reviewers. Any product that may be evaluated in this article, or claim that may be made by its manufacturer, is not guaranteed or endorsed by the publisher.
